# Particle Classification through the Analysis of the Forward Scattered Signal in Optical Tweezers

**DOI:** 10.3390/s21186181

**Published:** 2021-09-15

**Authors:** Inês Alves Carvalho, Nuno Azevedo Silva, Carla C. Rosa, Luís C. C. Coelho, Pedro A. S. Jorge

**Affiliations:** 1 Centre for Applied Photonics, INESC TEC, Rua do Campo Alegre 687, 4169-007 Porto, Portugal; nuno.a.silva@inesctec.pt (N.A.S.); carla.c.rosa@inesctec.pt (C.C.R.); luis.c.coelho@inesctec.pt (L.C.C.C.); pedro.jorge@inesctec.pt (P.A.S.J.); 2 Departamento de Física e Astronomia, Faculdade de Ciências da Universidade do Porto, Rua do Campo Alegre 687, 4169-007 Porto, Portugal

**Keywords:** optical tweezers, optical trapping, particle identification, Brownian motion, principal component analysis

## Abstract

The ability to select, isolate, and manipulate micron-sized particles or small clusters has made optical tweezers one of the emergent tools for modern biotechnology. In conventional setups, the classification of the trapped specimen is usually achieved through the acquired image, the scattered signal, or additional information such as Raman spectroscopy. In this work, we propose a solution that uses the temporal data signal from the scattering process of the trapping laser, acquired with a quadrant photodetector. Our methodology rests on a pre-processing strategy that combines Fourier transform and principal component analysis to reduce the dimension of the data and perform relevant feature extraction. Testing a wide range of standard machine learning algorithms, it is shown that this methodology allows achieving accuracy performances around 90%, validating the concept of using the temporal dynamics of the scattering signal for the classification task. Achieved with 500 millisecond signals and leveraging on methods of low computational footprint, the results presented pave the way for the deployment of alternative and faster classification methodologies in optical trapping technologies.

## 1. Introduction

First introduced in 1986 by Arthur Ashkin [1], optical tweezers (OT) explore optically-induced forces to capture, manipulate, and isolate micrometer-sized particles. This ability has made them increasingly popular in the field of biotechnology, where individual analysis of particles is crucial. Indeed, the technique can be applied from cellular studies, where it can be used to sort, identify, and manipulate single cells [2,3], to environmental sciences, for the identification of pollutants and aerosols [4], just to name a few examples. In particular, the accurate classification of the trapped specimen is a task of paramount importance, paving the way for OT-based systems that are able to enter into the mesoscopic scale and real-time operating devices.

The classification task of trapped particles has been shown over three major approaches. The first, arguably simpler, explores spatial image information, either using the image directly acquired from the microscope under external illumination [5,6] or using the spatial scattering pattern coming from the trapping laser itself [7]. In both situations, data analysis can be performed with a multitude of machine-learning approaches, benefiting from highly optimized algorithms for image classification such as convolution neural networks. The drawback of this approach is that the performance is strongly limited by the spatial resolution and acquisition rate of the optical imaging system, limiting its use to particles of a few micrometers and hinder the performance at real-time operation speeds.

A second common methodology combines optical tweezers with Raman spectroscopy, collecting and analyzing the spectral signature of the trapped particles [8]. Commonly referred in the literature as Raman tweezers, it can again leverage a multitude of machine-learning algorithms to extract relevant features from the spectrum collected and train the classification models [4]. This approach performs well for a wide range of applications, from detection of cancer in non-adherent blood cells to identification of single bacterial cells and assessment of its response to antibiotic treatment [8,9,10]. However, there is an increase in the cost of the setup due to the addition of the spectrometer. Furthermore, Raman analysis usually depends on relatively long integration times, decreasing the ability for high throughput.

More recently, a third approach sought trapped particle identification using the scattered signal signature in the temporal domain. The general concept rests on the fact that the signal scattered from trapped beads is related to their physical dynamics. This fact has been used for force calculation [11] and prediction [12], for probing Brownian motion [13,14], and for distinguishing trapped states even at the nanoscale regime [15,16]. For classification, an interesting approach was recently explored with flexible optical fiber tweezers [17,18] using the back-scattered signal obtained from trapped cells. Processing the data to extract a set of features both in the temporal and Fourier domain, a standard random forest algorithm allowed the discrimination of two gastric cancer cell models [19], demonstrating the potential of the technology for biomedicine applications such as early diagnosis and prognosis of cancer and neurodegenerative diseases [20].

Within this context, this work explores the classification of trapped beads from their scattering signal temporal dynamics, using a standard optical tweezers system equipped with a quadrant photodetector (QPD). It is proposed an alternative pre-processing strategy based on the Fourier transform of the acquired signal and subsequent principal component analysis, a methodology with a significantly lower computational load when compared with other previously reported feature extraction methods used in this topic of research [19]. The results suggest the possibility of correctly identifying the particle size and type with an accuracy around 90% with a signal of 500 milliseconds of duration which, to the best of our knowledge, is significantly smaller compared with previous time-based approaches [19,21].

## 2. Materials and Methods

### 2.1. Physical Model

Optical tweezers exploit light-induced forces generated by a tightly focused beam to trap and manipulate particles. The combination of the gradient force (associated with the spatial distribution of the optical intensity) and the scattering component (associated with the radiation pressure and photon momentum transference) creates an effective three-dimensional harmonic potential well that allows 3-D manipulation, provided the target specimen fulfils certain criteria regarding its refractive index and size [22,23].

Nevertheless, while the optical trap brings a trapped particle towards an equilibrium position, it still experiences a characteristic Brownian-type motion [22,23]. Indeed, from a microscopic perspective, an immersed particle is permanently moving in random directions due to the collisions with other molecules in the fluid [23,24]. The behavior of a trapped particle can be described as a dynamical equilibrium between random and deterministic contributions, expressed by the Langevin equation [23,25]

(1) $$m\ddot{r}\left(t\right)=-\gamma \dot{r}\left(t\right)-k_{p}⊙r\left(t\right)+\sqrt{2k_{B}T\gamma }W\left(t\right)$$

where r represents the position of the particle, *m* is the mass of the particle, γ is the particle friction coefficient, $k_{p}$ is the trap stiffness constant, ⊙ represents the Hadamard product, and W(t) is a stochastic term accounting for random collisions. Note the inclusion of the first term on the right-hand side, corresponding to a friction imposed by the medium, with a drag coefficient that can be approximated by the Stokes law of a spherical particle of radius *a* as:

(2) γ=6πηa,

where η is the viscosity of the medium. Typically, optical tweezers exploit systems of low Reynolds number, allowing us to drop the inertial term in Equation (1) and thus simplifying the model to its asymptotic behavior [23,25].

The existence of the Brownian motion allows us to experimentally probe physical properties of the trapped particle such as the optical force acting on it. Indeed, a wide range of passive methods (i.e. that do not require the action of an external force [26]) exist for computing the optical force, from which we can highlight two of the most commonly used: (i) the energy equipartition method and (ii) the power spectrum method.

The first method explores the equipartition theorem that states that, at thermal equilibrium, each degree of freedom contributes with $k_{B}T/2$, associated with the thermal fluctuations of the particle in a potential well, meaning that [22,27]:

(3) $$\frac{1}{2}k_{p}^{x}\langle {\left(x-x_{eq}\right)}^{2}\rangle =\frac{1}{2}k_{B}T,$$

with $k_{B}$ being the Boltzmann constant, $k_{p}^{x}$ the stiffness constant along the *x* axis, and *T* the absolute temperature. One of the drawbacks of the method is that precise information regarding the standard deviation of the position must be computed. This can be accomplished using the information of a QPD but requires prior calibration procedures [28,29].

The second method works around some of the drawbacks of the equipartition method by exploiting the evaluation of the dynamics in the frequency domain. At low Reynolds number, it can be shown that the one-sided power spectrum of the fluctuations of a trapped bead is given by the equation [22,27]:

(4) $$S_{xx}\left(f\right)=\frac{k_{B}T}{\pi^{2}\gamma \left(f_{0}^{2}+f^{2}\right)}$$

where $f_{0}$ corresponds to a particle-dependent corner frequency, related with the stiffness of the trap as:

(5) $$k_{p}^{x}=2\pi f_{0}\gamma .$$

By fitting the power spectrum to a Lorentzian function, the roll-off frequency can be easily extracted. This approach requires previous information regarding the particle radius as well as the drag coefficient to be known [27,28]. As such, the signal in the Fourier domain contains information regarding particle-specific behaviors and thus can in principle be explored for deploying a classification algorithm.

The temporal scattering signal depends on the particle characteristics, such as their size and refractive index [30]. With that in mind, different behaviors associated with distinct trapped particles can be exploited with a QPD.

### 2.2. Experimental Methods and Tools

**Figure 1:**
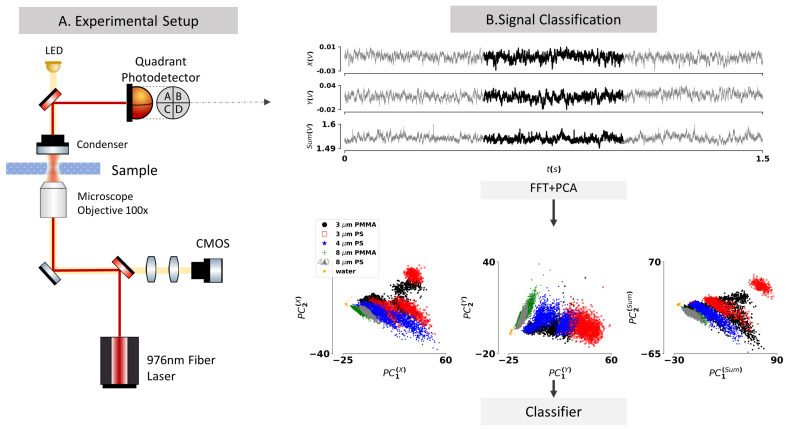
(**A**) Schematic of the optical tweezers system. (**B**) Schematic representation of the classification procedure, depicting the time scope of signals acquired from the quadrant photodetector (*X*, *Y*, SUM) and the PCA plots of the corresponding Fourier transforms for all the tested particles.

The optical tweezers system used in this work explores a conventional inverted microscope configuration (OTKB—Thorlabs modular optical tweezers system) for which schematic is provided in Figure 1A. The trapping laser, a fiber-coupled laser diode (Lumentum s27-7602-460) emitting at 976 nm, is coupled into the system through a Galilean beam expander. It is then focused by a 100× oil immersion objective to a focal spot of approximately 1.1 μm onto the sample, forming the optical trap. The transmitted laser is collimated by a condenser lens and directed towards a quadrant photodetector (PDQ80A-Thorlabs) placed at a conjugate back focal plane of the condenser. The detector is characterized by a wavelength range of 400–1050 nm, a bandwidth of 150 kHz, and the detector responsivity at 976 nm is approximately 0.65 (W/A). This type of detector yields a set of three signals (*X*, *Y*, SUM) that, upon calibration [22], may allow retrieving the *x* and *y* position of the particle scattered laser beam, along with the integral of the optical signal (SUM). Being sensitive to the displacement of the trapped particle in the transverse directions, the acquired signals reflect position fluctuations over time allowing to probe the Brownian motion of the trapped beads. Finally, to control the trapping procedure, an additional illumination system allows the imaging of the sample, with a standard 1280 × 1024 pixel color CMOS camera.

For testing the classification capabilities of the system, a set of reference particles (from Phosphorex) of different sizes and materials was used. The experimental measurements were conducted within aqueous solutions (deionized water, n = 1.3270 @976 nm [31]) of 0.05% polystyrene (PS) and polymethyl methacrylate (PMMA) microparticles with sizes ranging from 3 μm to 8 μm (Table 1). For each bead type, we acquired 6 distinct signals of 120 s of total duration at an acquisition rate of 10 kHz. These signals were employed to train and test a classification algorithm, which schematic is provided in Figure 1B.

### 2.3. Classification Algorithms and Procedures

The classification procedure proposed in this work aims to utilize the 3-channel signal acquired from the QPD (*X*, *Y*, SUM), to classify individual particles of different classes. As described above, the signal acquired is in principle related to the properties of the trapped particle through its dynamics, which may be sufficient to distinguish particles of distinct sizes and materials. As a test case, our goal was to discriminate between the different particle classes, presented in Table 1.

For the purpose of increasing the amount of data to train the model and approximate to a real-world scenario, each acquired signal (with a total duration of 120 s) was divided into individual segments of 500 milliseconds forming the total dataset to be classified. Then, for each signal and for each channel, was computed the Fourier transform using the fast Fourier transform (FFT) algorithm of the standard *numpy* library. Disregarding the continuum component, which does not contain information relative to the dynamics itself and thus can introduce unnecessary noise in the classifier, a principal component analysis (PCA) was applied using the *sklearn* library [33], keeping only the two most relevant principal components for each channel signal (see Appendix A). In short, these pre-processing procedures allow performing a dimensionality reduction from a signal of dimension 3 channels × 5000 data points into a final set of the 6 most relevant features of the co-variance space in the Fourier space, which by hypothesis are related to the properties of the particle dynamics and therefore can be used for deploying a final classification model.

To assess the performance of several classification algorithms and achieve the best results, a comprehensive set of algorithms exploiting the modular framework of the *sklearn* library were used. In particular, random forests (RF), support vector machines (SVM), k-nearest neighbors (KNN), and neural-network (NN) classifiers. The distinct algorithms were chosen to guarantee the diversity of the classification strategy employed and understand if any particular classifier is more suitable for this type of dataset. For each method, the hyper-parameter optimization was performed using a stratified 6-fold cross-validation procedure. For each fold, a subset containing all the signals corresponding to an individual particle of each type is left outside the training dataset and used only for the test dataset. Finally, the accuracy was computed using the same procedure after the hyper-parameter tuning procedure.

## 3. Results

The results obtained for the accuracy of each method are presented in Table 2, including the mean, the best, and the worst performance during the 6-fold cross-validation procedure. As can be seen, the performance of most of the methods is comparable, with KNN performing slightly better and RF slightly worse for the train–test dataset splits used. Furthermore, there is no significant variation of the performance across the validation folds, which suggest that the mean accuracy presented converges to a real-world scenario performance. These findings suggest that, for the classes of particles tested, the relations in the reduced PCA space can be easily identified by standard classification algorithms, demonstrating that it is possible to discriminate the particles using the implemented signal processing procedure.

**Figure 2:**
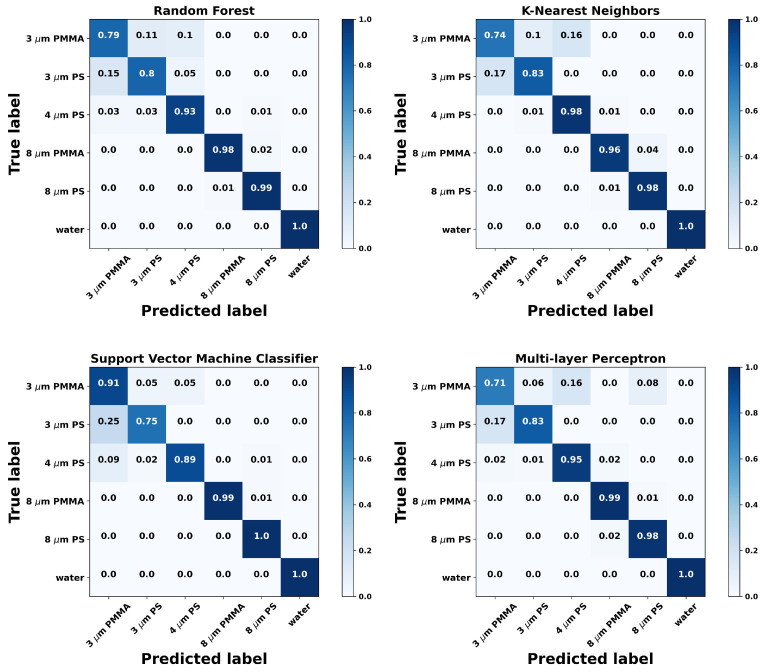
Confusion matrices showing the classification performance of the tested algorithms. The labels correspond to each particle type with the score corresponding to the mean accuracy obtained for the cross-validation procedure, as described in the main text.

Regarding the accuracy of the discrimination of particles itself, the confusion matrix for each algorithm was computed (see Figure 2), accumulating the results for each cross-validation fold and normalizing them at the end. The confusion matrix is useful to interpret the results, namely to understand the classifier performance for an individual class and understand possible reasons for its underperformance. As it can be appreciated, the classifier was able to successfully identify most of the classes. First, it can be observed that any algorithm can successfully identify all the situations that have no particle trapped, and correctly discriminate large and small-sized particles. Additionally, it is interesting to notice that at the 8 μm size, the model can discriminate between the two materials quite well, at around 98% accuracy level. Still, it can be seen that significant confusion appears for 3 μm PS and 3 μm PMMA particles, being also misidentified as 4 μm PS. The origin of this behavior may be either related to the proximity of the size of the particles, with some experimental uncertainty of their actual size during the fabrication procedure or even with the algorithm itself, something that shall be carefully evaluated in future studies with specifically designed experiments.

**Figure 3:**
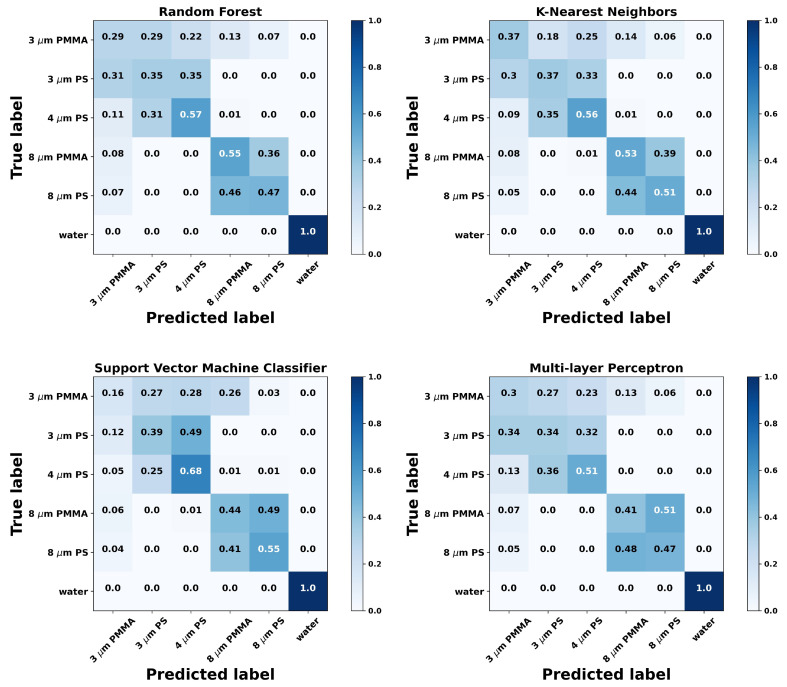
Confusion matrices showing the classification performance of the tested algorithms as in Figure 2. In this case, only the SUM channel of the QPD used.

Finally, it was also compared the performance of using all 3-channel information from the QPD against using only the SUM channel, which allows us to infer any benefits of using the QPD against a conventional photodetector for probing the front or back-scattered radiation. As it can be seen from Table 3 and Figure 3, the performance of using only the SUM channel is significantly lower, dropping to values just above 50%. Indeed, looking at the confusion matrices in Figure 3, it is straightforward to observe that while it can still differentiate between having a particle trapped or not, and between the small-sized particles and larger ones, the trained models are unable to discriminate the material types for similar sizes correctly. These findings suggest that a conventional OT system equipped with a fast response, position-sensitive QPD may outperform single detector scattering analysis methodologies previously reported in the literature [19].

## 4. Conclusions

This manuscript explored the possibility of classifying trapped particles on an optical tweezers setup by using scattered signal information from the trapping laser acquired by a quadrant photodetector. To perform dimensionality reduction and extraction of relevant features from the 3-channel time signal, a pre-processing procedure was introduced based on the transformation to the Fourier domain and subsequent principal components analysis. The processed data was then used to train and test various standard machine-learning classification models with a stratified cross-validation procedure. The results obtained suggest that this methodology can successfully discriminate a 6-class identification problem in controlled experimental conditions: polystyrene particles of 3 μm, 4 μm, and 8 μm, PMMA particles of 3 μm and 8 μm, and the case of no trapped particle. From the analysis of the confusion matrices, it can be concluded that the method is robust for most classes, allowing to discriminate particles not only in size but also in composite material. Furthermore, the lower accuracy obtained for the case when the SUM intensity channel alone suggests that the use of a quadrant photodetector may enhance the accuracy when compared against previously reported results that rely on the use of the full back-scattered signal [18,19].

Overall, compared with the previous literature on the topic [18,19], we shall highlight that the methodology hereby proposed requires smaller signal intervals, around 500 milliseconds, and features a simpler feature extraction procedure with a lower computational load. Therefore, the results not only validate the capability of the standard optical tweezers system equipped with QPD but also pave the way for significantly faster operation rates towards real-time monitoring applications, enabling a new set of applications for which real-time performance is paramount.

To finalize, we can also put the work in a broader perspective by discussing it at the application level. Indeed, while multiple experimental techniques are known capable of identifying micrometer size particles, the features of the OT systems offer an array of unique possibilities. For example, when compared with systems based on flow cytometry or electrodynamic balance [34,35,36], OT presents itself as an integrated technological solution that is capable to perform simultaneously the identification and manipulation of individual particles. In this context, the ability to perform both sensor and actuator functionalities with a single optical tool makes it more suitable for integration into optofluidic chips, thus being an enabler for future technological solutions on challenges such as point of care diagnostic, liquid biopsies, and process control in bioreactors.

## Figures and Tables

**Table 1:** Materials and optical characteristics of the particles and solution used in the experimental measurements.

Particle Type	Particle Size	Refractive Index (@976 nm)
Polystyrene	3 μm	
microspheres	4 μm	1.5731 [32]
(PS)	8 μm	
Polymethyl	3 μm	
Methacrylate	8 μm	1.4824 [32]
(PMMA)		

**Table 2:** Performance results for various classification algorithms obtained for the test datasets of the cross-validation procedure described in the main text.

Method	Accuracy—Test Dataset		
	Mean	Best	Worst
Random Forests	0.91	0.99	0.77
Support Vector Machines	0.92	0.98	0.74
K-Nearest Neighbours	0.91	0.99	0.77
Multi-layer Perceptron	0.91	0.99	0.69

**Table 3:** Performance results for various classification algorithms obtained using only the SUM channel from the QPD.

Method	Accuracy—Test Dataset		
	Mean	Best	Worst
Random Forests	0.54	0.64	0.45
Support Vector Machines	0.53	0.64	0.42
K-Nearest Neighbours	0.55	0.67	0.44
Multi-layer Perceptron	0.49	0.61	0.41

**Figure A1:**
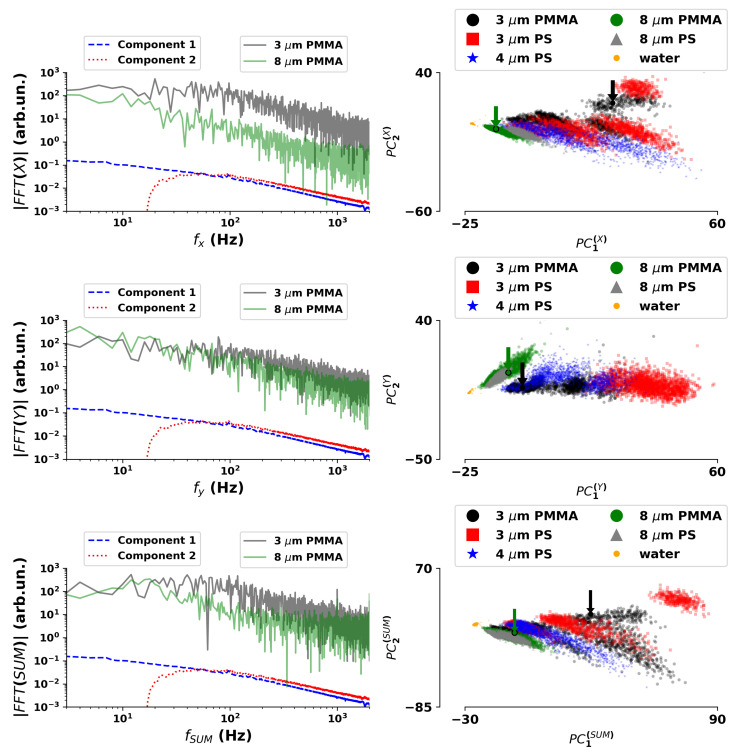
Results of the PCA analysis of Channels *X*, *Y* and SUM. Left-hand side plots depict the first two principal components obtained for the PCA analysis as well as a typical signal obtained for two distinct particles for the 3 distinct channels. Subfigures on the right-hand side display the results of the PCA analysis as a two-dimensional plot with the first two principal components as the extracted features. Additionally represented and highlighted by the arrows are the signals for the particles represented on the left-hand side plots.

**Figure A2:**
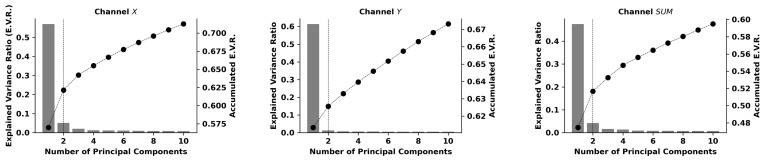
Scree plot for the PCA analysis presented in Figure A1, displaying the explained and accumulated variance ratio in function of the principal components used.

## Data Availability

The data used in this project as well as a version of the algorithm are available upon request.

## Abbreviations

The following abbreviations are used in this manuscript:

PS	Polystyrene
PMMA	Polymethyl Methacrylate
OT	Optical Tweezers
QPD	Quadrant Photodetector
PCA	Principal Component Analysis
FFT	Fast Fourier Transform

## Appendix A. Notes on the Pre-Processing Procedure

The methodology employed in this work made use of a principal component analysis during the pre-processing procedure. As briefly introduced in the main text, the option for this unsupervised data analysis methodology has two major purposes, discussed in this appendix in more detail.

The first objective of performing the PCA was to extract a set of relevant features that was capable of describing the information gathered from the scattering signal of the position fluctuations of a particle in the Fourier domain. Acting in the co-variance space formed by all acquired signals, the PCA algorithm seeks the linear combinations of variables in this initial high-dimensional space which are associated with the largest amount of the variance of the data. Putting it in context with our work, the procedure extracts a tendency along the frequency space that is suitable to describe the variation of the Fourier transform observed for the total set of signals.

The second objective of the procedure was to perform a dimensionality reduction and thus avoid the curse of dimensionality [37]. Indeed, the segment to classify has a total of 3channels×5000datapoints = 15,000 variables, an impracticable number of features to deploy a standard classifier due to sparsity issues that usually occurs in data of such high-dimensionality. To avoid this curse of dimensionality, we performed a dimensionality reduction by keeping only the information associated with most relevant components of the PCA. Furthermore, this comes with an additional advantage, as the PCA analysis also proves helpful to deal with the stochastic noise characteristic of the measures. Indeed, this projection into a final space of reduced dimensionality plays a similar role of a filter [38], thus working around the stochastic noise of the data.

All these remarks can be observed and confirmed in the graphical display of the results of the PCA algorithm presented in Figure A1. First, on the left-hand side, it can be noticed that while a typical signal is extremely noisy, the first two principal components are smooth lines across the frequency domain, which indicate the filter capacity of the PCA algorithm. Plots on the right-hand side show that each channel provides some degree of discrimination between distinct classes, which can be combined and used for deploying the classification algorithm. Finally Figure A2 presents the scree plot for the PCA analysis of Figure A1, showing the explained variance ratio in function of the number of components. Clearly the largest amount of variation is concentrated in the two first principal components, with the remaining variance equally distributed by the other components, most possibly accounting for the noisy part of the data. This observation alone does not warrant that the best set of features to separate the data is being used but is a strong suggestion of the validity of the dimensional reduction procedure, a fact that is confirmed by the performance of our algorithm as presented in the manuscript.
